# Peroxiredoxin 3 maintains the survival of endometrial cancer stem cells by regulating oxidative stress

**DOI:** 10.18632/oncotarget.21580

**Published:** 2017-10-06

**Authors:** In-Sung Song, Yu Jeong Jeong, Young Jin Seo, Jung Mi Byun, Young Nanm Kim, Dae Hoon Jeong, Jin Han, Ki Tae Kim, Sung-Wuk Jang

**Affiliations:** ^1^ Department of Biomedical Sciences, College of Medicine, Ulsan University, Asan Medical Center, Seoul, Republic of Korea; ^2^ Department of Obstetrics & Gynecology, Paik Institute for Clinical Research, Busan Paik Hospital, Inje University, Busan, Republic of Korea; ^3^ National Research Laboratory for Mitochondrial Signaling, Department of Physiology, College of Medicine, Cardiovascular and Metabolic Disease Center, Inje University, Busan, Republic of Korea

**Keywords:** peroxiredoxin 3, endometrial cancer, cancer stem cells, oxidative stress, mitochondria

## Abstract

Cancer stem cell (CSC)-targeted therapy could reduce tumor growth, recurrence, and metastasis in endometrial cancer (EC). The mitochondria of CSCs have been recently found to be an important target for cancer treatment, but the mitochondrial features of CSCs and their regulators, which maintain mitochondrial function, remain unclear. Here, we investigated the mitochondrial properties of CSCs, and identified specific targets for eliminating CSCs in EC. We found that endometrial CSCs displayed higher mitochondrial membrane potential, Ca^2+^, reactive oxygen species, ATP levels, and oxygen consumption rates than non-CSCs. Further, we also verified that mitochondrial peroxiredoxin 3 (Prx3) was upregulated, and that it contributed to the survival of CSCs in EC. The knockdown of the Prx3 gene resulted not only in decreased sphere formation, but also reduced the viability of endometrial CSCs, by causing mitochondrial dysfunction. Furthermore, we found that the forkhead box protein M1 (FoxM1), an important transcriptional factor, is overexpressed in patients with EC. FoxM1 expression correlates with elevated Prx3 expression levels, in agreement with the tumorigenic ability of Prx3 in endometrial CSCs. Taken together, our findings indicate that human endometrial CSCs have enhanced mitochondrial function compared to that of endometrial tumor cells. Endometrial CSCs show increased expression of the mitochondrial Prx3, which is required for the maintenance of mitochondrial function and survival, and is induced by FoxM1. Based on our findings, we believe that these proteins might represent valuable therapeutic targets and could provide new insights into the development of new therapeutic strategies for patients with endometrial cancer.

## INTRODUCTION

Endometrial cancer (EC) is commonly diagnosed at an early stage, as a tumor originating in the endometrium. EC can be classified into two subtypes based on its histological characteristics, grade, and hormone receptor expression—endometrioid (type I) and non-endometrioid (type II) EC [1]. In particular, type II ECs are known to show poor prognosis and have a higher risk of metastasis. Type II ECs include a range of histological subtypes, each showing distinct molecular and genomic features, such as serous, carcinosarcoma, and clear cell ECs [2, 3] and account for 10–15% of the ECs, but cause 40% of EC-related deaths because of the high incidence of associated extrauterine diseases, especially lymph node metastases [4, 5]. Moreover, the recurrence rate of EC is about 15%, with more than half of the cases of recurrence occurring within two years of primary treatment; the recurrence rate can also reach a value of 50% in aggressive histologies (non-endometrioid ECs; [6, 7]). For women with metastatic or aggressive ECs, the predominant treatment is endocrine therapy or chemotherapy. However, the prognosis for these patients is poor. Cases with high-grade aggressive and metastatic ECs show poor clinical outcomes because of the lack of targets required for targeted therapy. Drugs such as cisplatin, carboplatin, paclitaxel, and doxorubicin have been used in single-agent regimens for EC treatment, and showed a response rate ranging from 21% to 36% in patients with EC [8]. Together, these data indicate the need for new therapies based on a better understanding of the molecular mechanisms underlying EC, its recurrence, and its metastasis.

Recently, cancer stem cell theory has garnered a lot of research interest, mainly due to the existence of a relatively rare, highly drug resistant, quiescent population of tumor initiating cells known as cancer stem cells (CSCs). CSCs are maintained by self-renewal and give rise to differentiated tumors via a multifactorial process; they show elevated expression of genes involved in glycolysis, and alterations in oxidative phosphorylation [9]. Several studies have reported the presence of CSCs in EC, and that of CD133 as a marker of endometrial CSCs [10, 11]. Moreover, it has been reported that mitochondrial activity and reactive oxygen species (ROS) regulate the stemness of stem cells, and the relationship between mitochondrial function and pluripotency has also been proven [12–14]. Mitochondria play important roles in cell death through various mechanisms, aside from their role in energy production [15–17]. Specifically, one mitochondrial feature consists of a multi-level network of redox defense systems for the elimination of hydrogen peroxide, generated in the oxidative phosphorylation system, as a consequence of ATP production. As antioxidant proteins in the mitochondria, peroxiredoxin 3 (Prx3), peroxiredoxin 5, superoxide dismutase 2, and thioredoxin 2 eliminate ROS that have been generated in the oxidative phosphorylation system [18, 19]. Thus, the inhibition of antioxidant proteins may provide a targeted therapy that leads to mitochondrial dysfunction and cell death.

Recently, the mitochondrial features of CSCs in diverse types of cancer such as lung cancer, ovarian cancer, and glioblastomas have been reported [20–22]. Specifically, we have reported the mitochondrial energy metabolism-related properties of colon CSCs [23], and showed that colon CSCs utilize the primary mitochondrial oxidative phosphorylation pathway, rather than glycolysis, to produce ATP. Moreover, Prx3 was identified as a regulator of colon CSCs. Mitochondrial Prx3 is overexpressed in hepatocellular carcinoma [24] and breast cancer [25]. Prx3 was also found to be reduced in the brain of patients with Alzheimer's and Down syndrome [26]. Furthermore, it was reported that Prx3 was overexpressed in EC [27]. However, the role of Prx3 and the mitochondrial features of endometrial CSCs have not been clearly defined so far. It is also necessary to identify possible therapeutic targets for the treatment of aggressive or metastatic ECs using mitochondrial-specific targets of CSCs in other tissues. In this study, we aimed to investigate the mitochondrial features of CSCs, and successfully identified specific target proteins that can be used in the targeted therapy of EC in the future.

## RESULTS

### Endometrial CSCs utilize the mitochondrial oxidative phosphorylation system to produce energy

**Figure 1:**
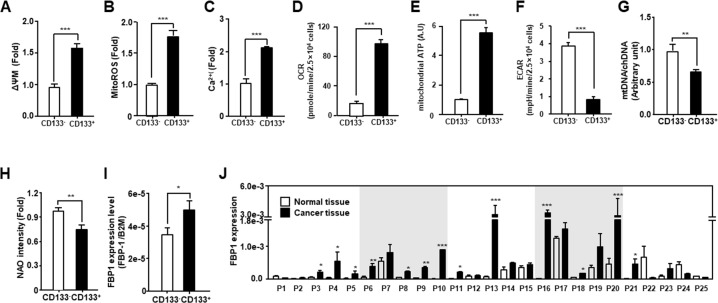
Comparison of mitochondrial function in endometrial CSCs (CD133+) and non-CSCs (CD133−) **(A-F)** Mitochondrial membrane potential (A), mitochondrial ROS levels (B), calcium levels (C), OCR (D), mitochondrial ATP levels (E), and ECAR (means relative to lactic acid production (F) measured in CD133^+^ and CD133^−^ sorted cells from the Ishikawa endometrial cancer cell line. ECAR, extracellular acidification rate. Mitochondrial ATP levels are measured by quantifying the luciferase-catalyzed, ATP-dependent oxidation of luciferin in endometrial CSCs and non-CSCs. **(G)** Mitochondrial DNA content calculated from the ratio of mitochondrial DNA to chromosomal DNA, measured by qPCR using primers specific to the mitochondrial D-loop, and chromosomal β2-microglobulin (*B2M*) gene. **(H)** Mitochondrial content measured by fluorescence-activated analysis in CD133^+^ and CD133^−^ cells stained with nonyl acridine orange (NAO) dye. **(I)** qRT-PCR analysis of *FBP1* levels, which are related to glycolysis and gluconeogenesis, in CD133^+^ and CD133^−^ cells isolated from Ishikawa cells. **(J)** Transcript levels for *FBP1* in 25 pairs of tissues from human patients with EC, measured by qRT-PCR. *FBP1* levels are calculated using standard methods, after normalizing against the *B2M* level in each sample.

We evaluated the mitochondrial function of endometrial CSCs (CD133^+^) compared to that of non-CSCs (CD133^−^) isolated using a CD133 antibody (Figure 1). CD133^+^ cells had a higher mitochondrial membrane potential (ΔΨm) and increased levels of ROS and Ca^2+^, compared to those measured in CD133^−^ cells (Figure 1A-1C). Moreover, the oxygen consumption rate (OCR) and mitochondrial ATP levels of CSCs were higher than those of non-CSCs. In contrast, lactate production was lower in CSCs than in non-CSCs (Figure 1D-1F). Next, we compared the mitochondrial DNA level and total mitochondrial content between endometrial CSCs and non-CSCs. As shown in Figure 1G-1H, CSCs presented less mitochondrial DNA than non-CSCs, and showed a lower fluorescence intensity of the mitochondrial content, as measured by nonyl acridine orange staining. Finally, we assessed the level of the fructose 1,6-biphosphatase (FBP1) gluconeogenic enzyme, which has been shown to contribute to the characteristics of CSCs in basal-like breast cancer [28] and found that the level of FBP1 was higher in CSCs than in non-CSCs (Figure 1I). Moreover, the FBP1 level was increased by more than 2-fold in more than 60% of the EC tissues, compared to that in normal tissues from patients with EC (Figure 1J). These data suggest that endometrial CSCs display enhanced mitochondrial functions, such as an increased OCR, and elevated ATP production.

### Mitochondrial Prx3 shows higher expression in endometrial CSCs than in non-CSCs

**Figure 2:**
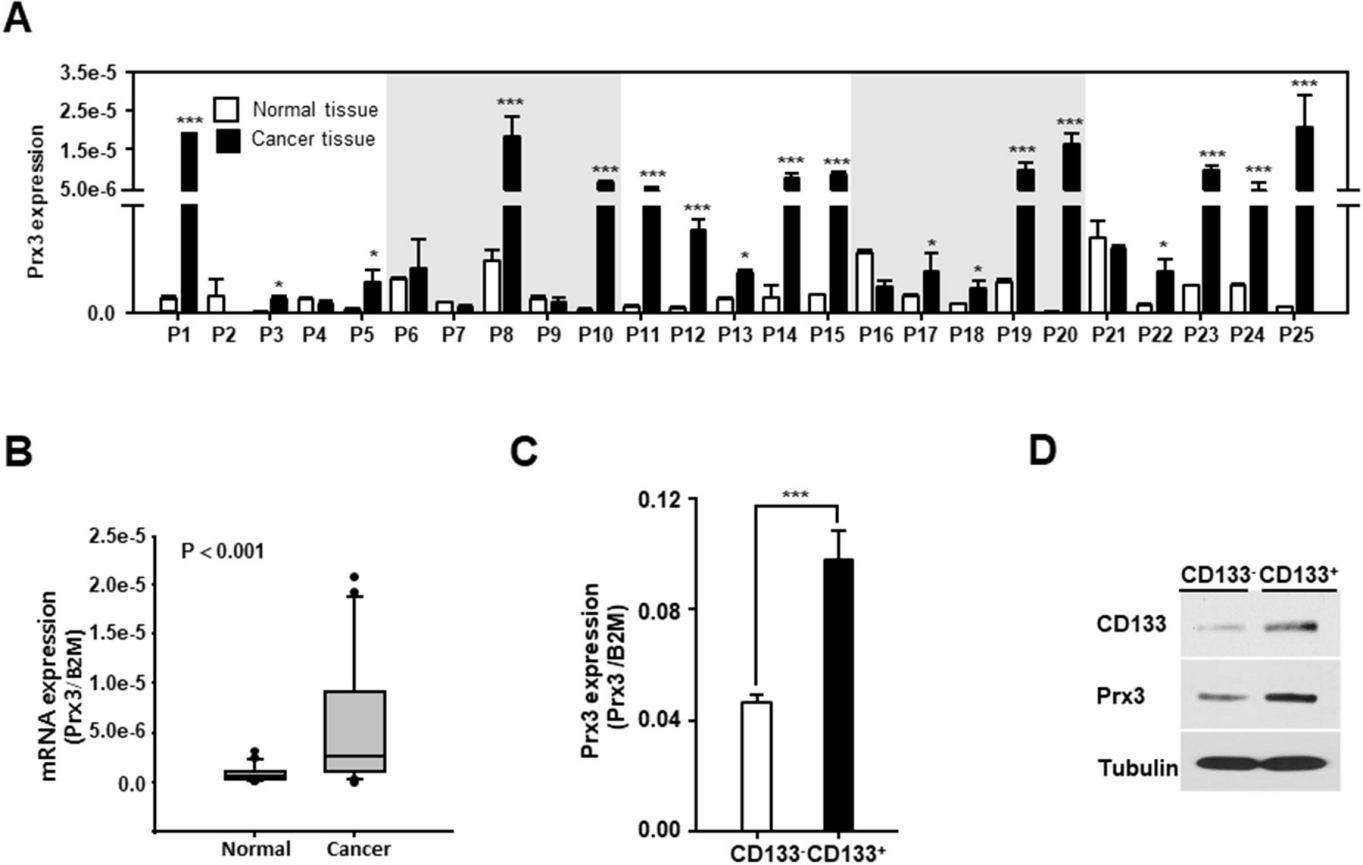
Mitochondrial Prx3 is upregulated in CD133+ cells and human EC tissues **(A and B)** Transcript levels for Prx3 in 25 pairs of tissues from human patients with EC, measured by qRT-PCR (A). The box plot analysis shows the median and 25^th^ and 95^th^ percentiles, based on the results from Figure 2A (B). **(C and D)** Prx3 expression, measured using a qRT-PCR (C) and western blotting (D) in the CD133^+^ and CD133^−^ subpopulations, isolated from Ishikawa cells.

Next, we aimed to identify the potential regulators of mitochondrial activity, which lead to stemness and anticancer drug resistance and metastasis. It was recently reported that Prx3 is highly expressed in patients with EC [27], but the function of Prx3 in EC and endometrial CSCs has not been clearly defined. To examine whether Prx3 is involved in mitochondrial activity, we first confirmed the expression of Prx3 in patients with EC. As shown in Figure 2A and 2B, Prx3 mRNA expression was higher in EC tissues than in normal endometrial tissues. Moreover, we observed that Prx3 expression was higher in the CD133^+^ cell population than that in the CD133^−^ cell population that was isolated from Ishikawa EC cells (Figure 2C and 2D), suggesting that Prx3 may play a critical role in the mitochondrial function of endometrial CSCs, and in the carcinogenesis of the endometrium.

### Prx3 depletion results in the death of endometrial cancer cells by causing mitochondrial dysfunction

**Figure 3:**
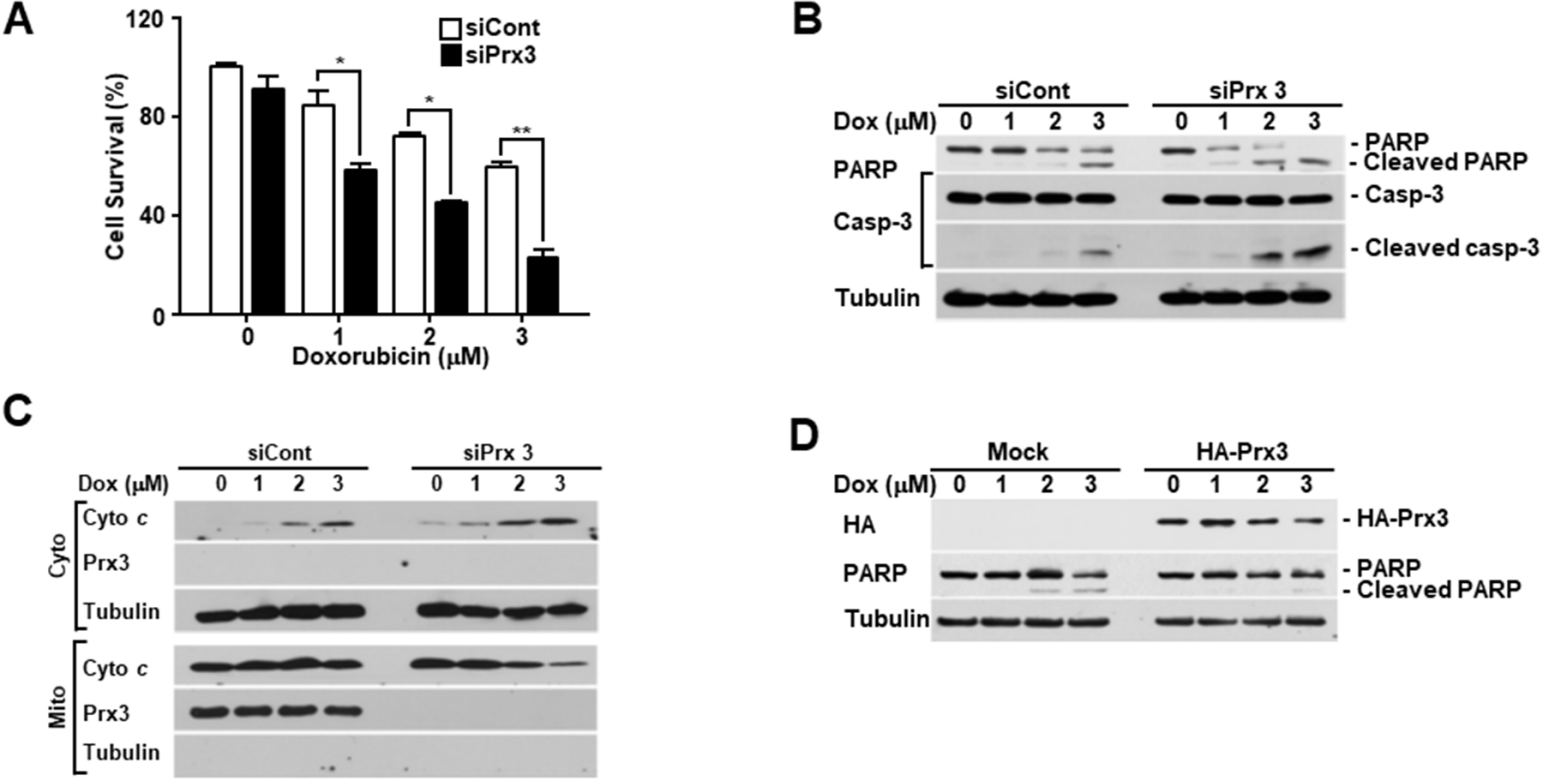
Prx3 regulates doxorubicin-induced cell death in EC cells **(A-C)** Ishikawa cells transfected with siRNA against Prx3 or control siRNA, and treated with the indicated dose of doxorubicin for 24 hours. (A) Doxorubicin-induced cell death measured using FACSCantoII analysis, after staining using an annexin V-fluorescein isothiocyanate (FITC)/7-amino actinomycin D (7-AAD) staining kit. (B) Western blotting (with the indicated antibodies) of Prx3-depleted cells, treated with doxorubicin at the indicated doses. (C) Western blotting of cells transfected with siCont/siPrx3, treated with doxorubicin for 24 hours, and separated into cytosolic and mitochondrial fractions. Tubulin and Prx3 antibodies are used as markers of the cytosolic and mitochondrial fractions, respectively, and cytochrome *c* is used to indicate mitochondrial-mediated cell death. **(D)** Poly ADP-ribose polymerase and tubulin levels in Ishikawa cells transfected with pHA-Prx3 or Mock plasmid, followed by treatment with doxorubicin for 24 hours at the indicated dose.

**Figure 4:**
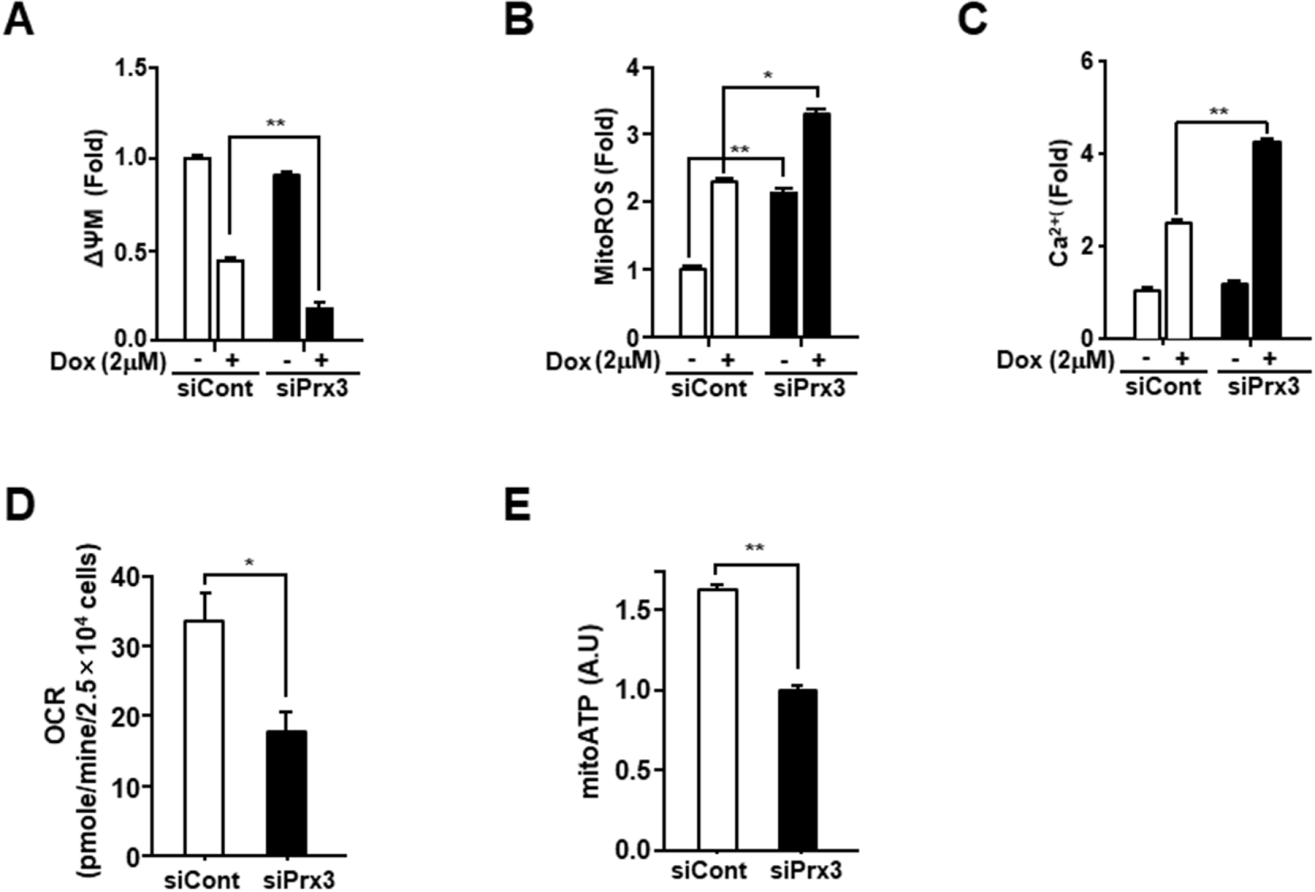
Prx3 knockdown regulates mitochondrial activity in EC cells **(A-C)** Ishikawa cells transfected with siPrx3 or siCont for 48 hours, and then treated with doxorubicin or vehicle for 24 hours. Mitochondrial membrane potential (A), mitochondrial ROS levels (B), and calcium levels (C) measured using FACSCantoII analysis, after staining with TMRE, Mito-Sox, and Rhod-2AM. **(D and E)** Ishikawa cells transfected with siRNA against Prx3 or siCont; OCR (D) and mitochondrial ATP levels (E).

Doxorubicin is a commonly used as an anticancer drug in endometrial carcinoma [29]. To explore the role of Prx3 in doxorubicin-induced cell death, we conducted an *in vitro* cell death assay using annexin V-FITC/7-AAD in doxorubicin-treated Ishikawa cells, which were transfected with siRNA to deplete Prx3. As shown in Figure 3A, the use of siPrx3 led to increased cell death, compared to that achieved using control siRNA, which was dependent on the dosage of doxorubicin. Next, we used immunoblot analysis to determine whether Prx3 depletion modified caspase-3 and poly (ADP-ribose) polymerase (PARP) in a dose-dependent manner in doxorubicin-treated cells. The cleaved bands of caspase-3 and PARP were more intense in lysates from Prx3-depleted cells, than in lysates from control cells (Figure 3B). Furthermore, we examined whether mitochondria are involved in the doxorubicin-induced cell death, following Prx3 depletion. In our experiments, the release of cytochrome *c* was markedly increased in the cytosol of Prx3-depleted cells compared to that of siRNA-transfected control cells (Figure 3C). On the other hand, immunoblot analysis in Prx3-overexpressed cells were shown to decrease cleavage of PARP by doxorubicin treatment (Figure 3D). These results suggest that the mitochondrial dysfunction caused by Prx3 depletion plays a critical role in cell death caused by the direct activation of the caspase cascade following doxorubicin treatment. To further investigate whether Prx3 regulates mitochondrial activity, we investigated the mitochondrial features of Prx3-depleted cells. ΔΨm levels were lower in the Prx3-depleted cells compared to those in control cells (Figure 4A). We next measured the mitochondrial ROS and Ca^2+^ levels by using Mito-Sox, an oxidant-sensitive fluorescent dye, and rhod2-AM, a mitochondrial Ca^2+^-sensitive dye, respectively. Prx3 depletion resulted in a remarkable increase in mitochondrial superoxide anion production and in the Ca^2+^ levels (Figure 4B and 4C). To better characterize the mitochondrial energy metabolism underlying the effect of Prx3 depletion, we analyzed the OCR and ATP production in these cells, and found that the basal levels of oxygen consumption (Figure 4D) and ATP production (Figure 4E) were significantly decreased in Prx3-depleted cells compared to those in control cells. Taken together, our results suggest that the alterations in mitochondrial function caused by Prx3 depletion are involved in the susceptibility of endometrial cancer cells to doxorubicin-mediated cell death.

### Mitochondrial Prx3 contributes to the maintenance of stemness and the survival of endometrial CSCs

**Figure 5:**
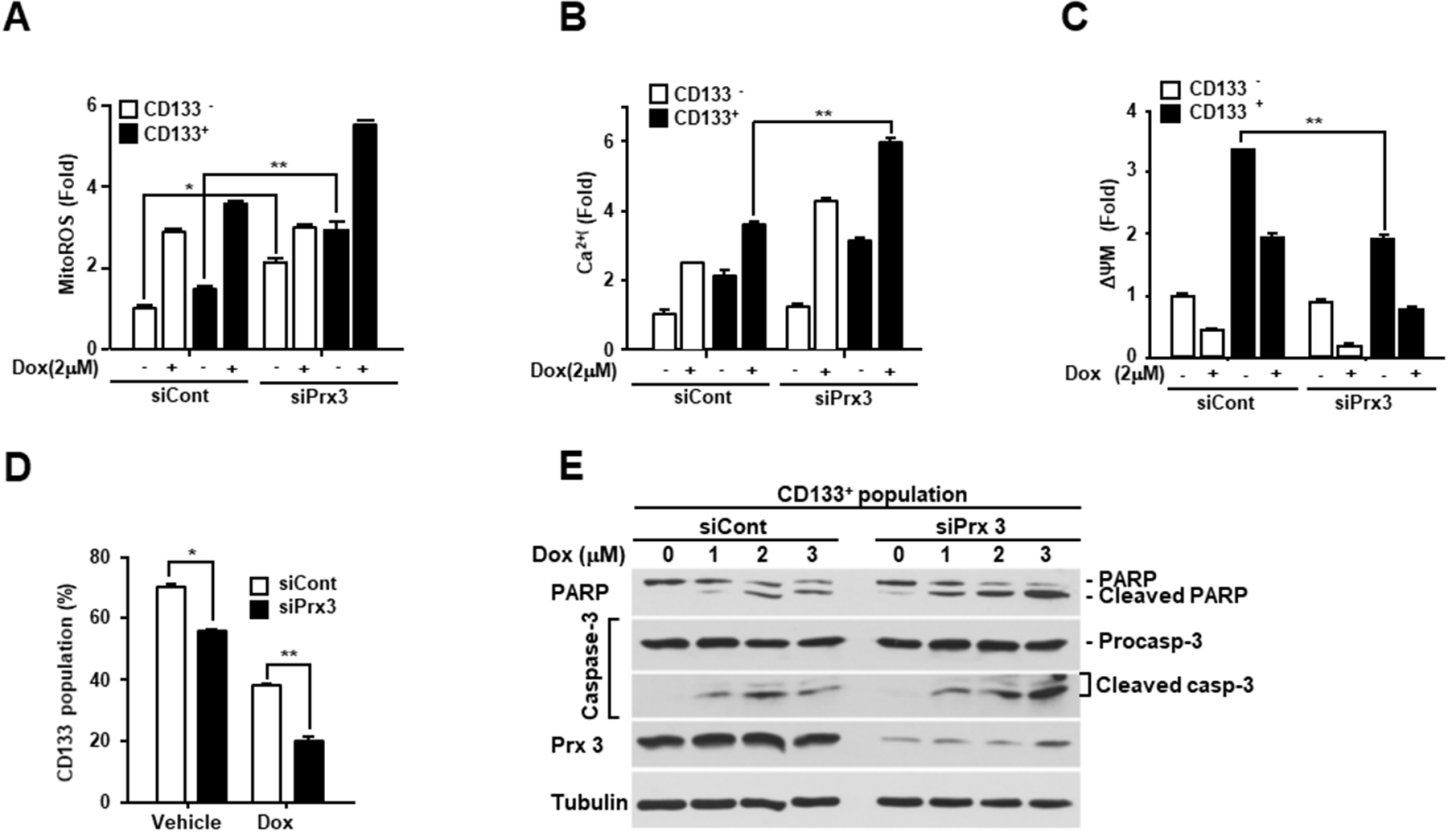
Prx3 depletion induces mitochondrial dysfunction and inhibits the CSC population **(A-D)** Ishikawa cells transfected with siPrx3 or siCont, and treated with doxorubicin for 24 hours. The mitochondrial ROS levels (A), calcium level (B), and mitochondrial membrane potential (C) as measured in CD133^+^ and CD133^−^ cells. The CD133^+^ population, analyzed using a flow cytometer, in the siPxr3-transfected Ishikawa cells (D). **(E)** Doxorubicin-treated CD133^+^ cells, isolated from the Prx3-depleted and control Ishikawa cells, respectively. Western blots with antibodies against PARP, caspase-3, Prx3, and tubulin are shown.

To determine the role of Prx3 in endometrial CSCs, we isolated CSCs from Prx3-depleted and control cells using an anti-CD133 antibody, and observed a significant increase in mitochondrial ROS levels in the both the Prx3-depleted CSCs and non-CSCs (Figure 5A). In contrast, the Ca^2+^ levels slightly increased in the Prx3-depleted CSCs (Figure 5B). While there was no change in the ΔΨm after Prx3 depletion in non-CSCs, the ΔΨm in CSCs was significantly decreased by Prx3 depletion (Figure 5C). Thus, the ΔΨm of CSCs decreased concomitantly with an increase in ROS and Ca^2+^ levels upon Prx3 depletion, but this effect was not observed in non-CSCs, which suggested that endometrial CSCs were more dependent on the mitochondrial oxidative phosphorylation system for ATP production than endometrial non-CSCs. Moreover, the basal ROS level in CSCs was further increased upon doxorubicin treatment, and resulted in a further decrease in the ΔΨm of CD133^+^ cells, an effect that was not observed in endometrial non-CSCs. In fact, the endometrial CSC population in the Prx3-depleted cells was also significantly decreased, which indicated mitochondrial dysfunction (Figure 5D). The effect of the doxorubicin treatment was also clearly more pronounced in the Prx3-depleted cells than that in the controls. Consistent with this observation, Prx3 depletion in endometrial CSCs also intensified the cleavage of procaspase-3 and PARP, which was dependent on the dosage of doxorubicin (Figure 5E).

### Prx3 regulates endometrial CSC-mediated tumorigenesis

**Figure 6:**
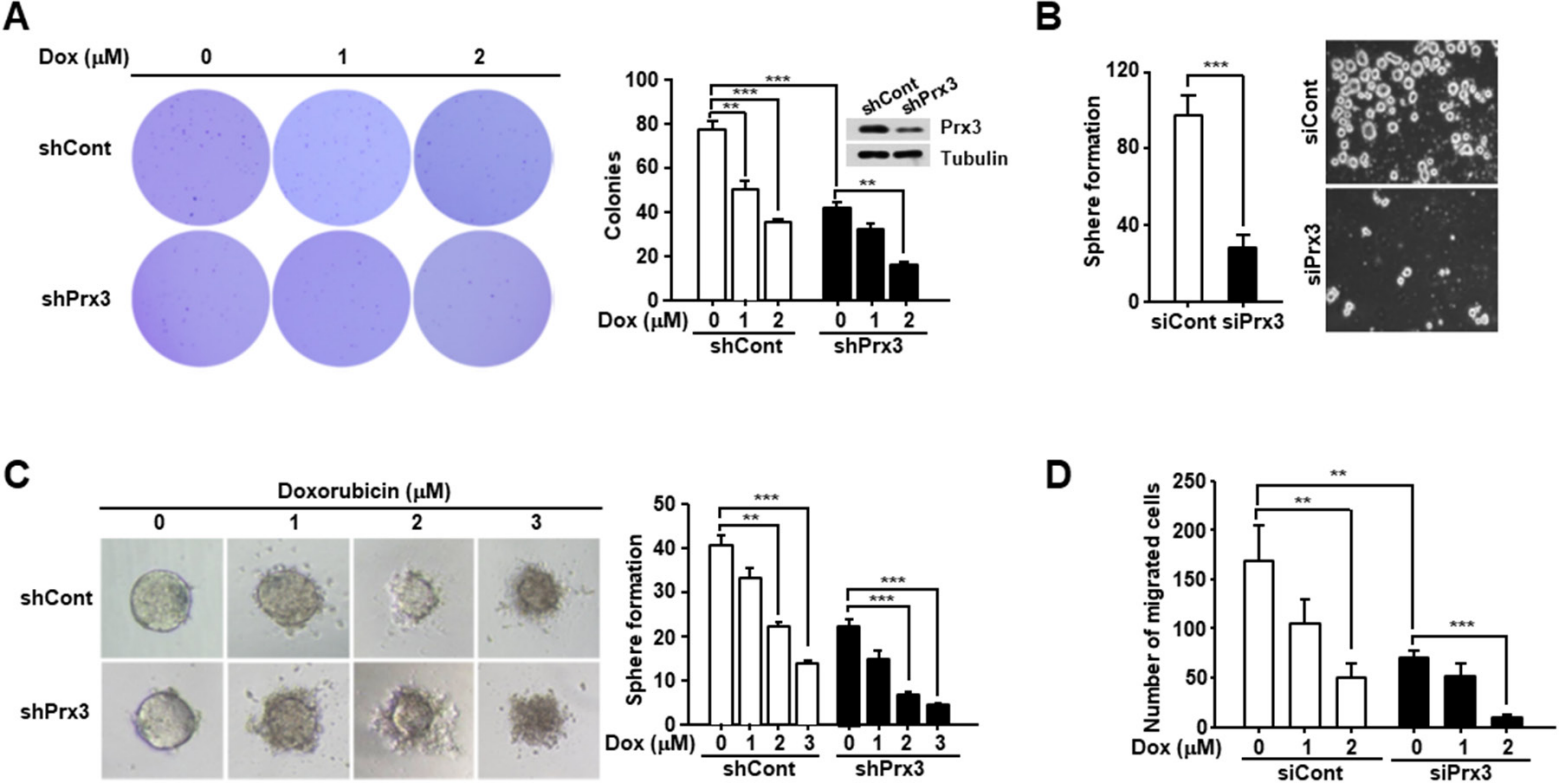
Prx3 depletion decreases the tumorigenic ability of Ishikawa cells **(A)** Prx3 shRNA and control shRNA were transfected into Ishikawa cells. These cells were seeded onto soft agar, and after 24 hours, the cells were treated with doxorubicin, which was changed every five days. The number of colonies generated per 10,000 cells was counted three weeks later. **(B)** Prx3-depleted and control cells, subjected to sphere formation assays in ultra-low-attachment 6-well plates. The number of spheroids generated per 10, 000 cells was counted one week later. **(C)** Prx3-depleted and control cells plated in ultra-low-attachment plates. The generated spheroids were seeded at a single spheroid per well into 50 wells of ultra-low-attachment 96-well plates. Representative photomicrographs show the shapes of the spheroids after treatment with doxorubicin for 24 hours. The unbroken spheroids were counted and represented in Figure 6C (right panel). **(D)** Prx3-depleted and control Ishikawa cells were allowed to migrate through a transwell for 24 hours. Cells were stained and visualized by microscopy using a x10 objective. Number of migrated cells were counted in six randomly selected fields per well.

To examine the effect of Prx3 depletion on the modulation of endometrial CSC-mediated tumorigenesis, we performed an anchorage-independent growth assay. Prx3-depleted cells showed a significant reduction in colony formation compared with control cells (Figure 6A). We also treated the cells with doxorubicin, as indicated in Figure 6A, and observed a reduced number of colonies in the Prx3-depleted cells compared to that in the control cells. Furthermore, we performed a sphere formation assay, and found that sphere formation was reduced by approximately 80% in Prx3-depleted cells compared to that in control cells (Figure 6B). In addition, the spheres formed in the Prx3-depleted cells showed enhanced sensitivity to doxorubicin treatment in a dose-dependent manner, compared with those in control cells (Figure 6C). Finally, we performed the migration test, and Prx3-depleted Ishikawa cells decreased the number of migrated cells compared with control cells. Moreover, the effect of Prx3-depletion dramatically increased by the combination treatment with doxorubicin (Figure 6D). These data suggest that Prx3 depletion sensitized endometrial CSCs to the doxorubicin-induced cell death, by causing mitochondrial dysfunction.

### Prx3 expression by FoxM1 regulates its effect on the survival of endometrial CSCs

**Figure 7:**
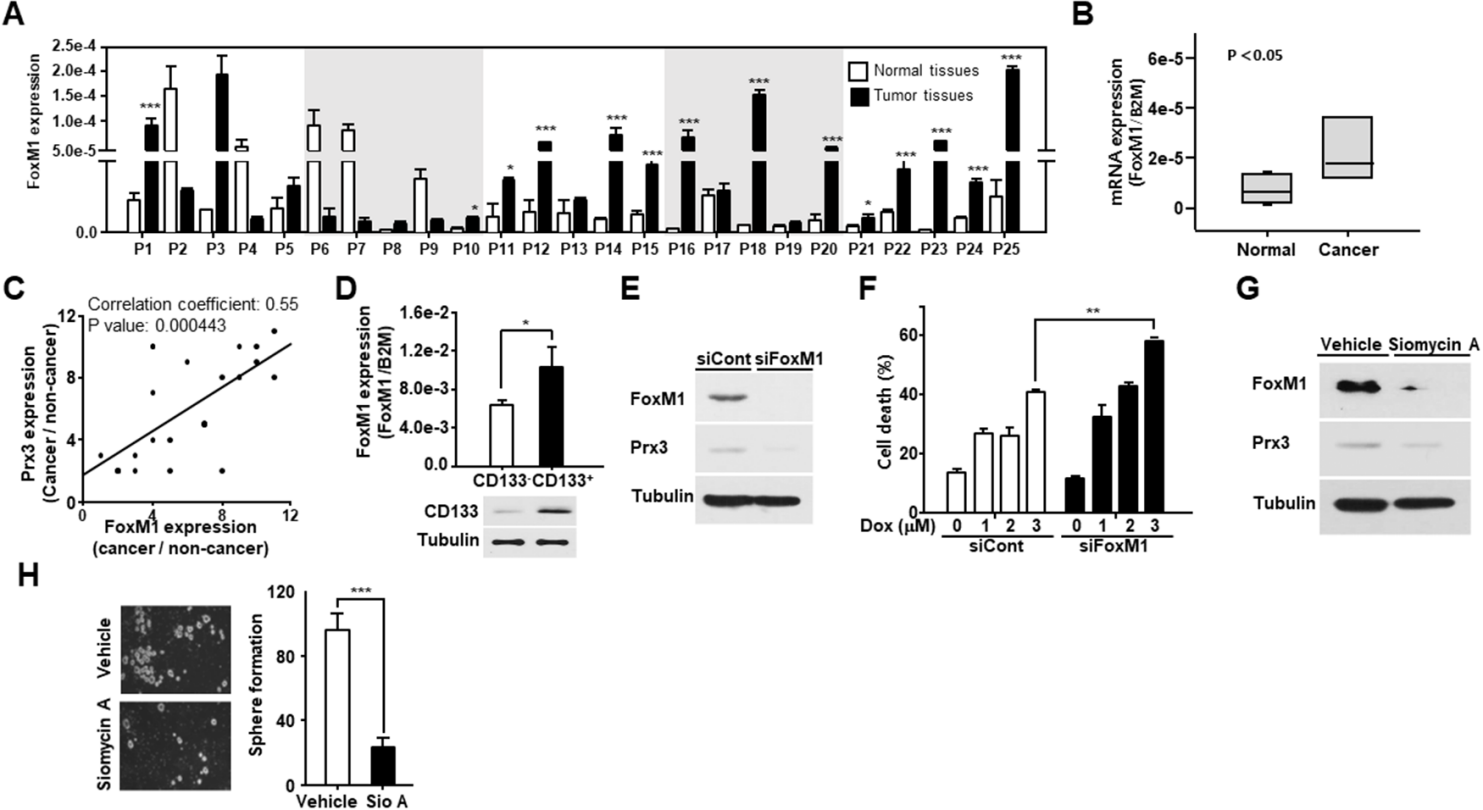
FoxM1-induced Prx3 gene expression regulates the survival of endometrial CSCs **(A and B)** Transcript levels for *FOXM1* in 25 pairs of tissues from human patients with endometrial cancer, measured by qRT-PCR (A). The box plot analysis shows the median, and the 25^th^ and 95^th^ percentiles, based on the results from Figure 7A (B). **(C)** Comparison of *FOXM1* and Prx3 expression in tissues of patients with EC. Corresponding correlation coefficients and *P* values are shown. **(D)** Using a CD133 antibody, sorted CD133^+^ and CD133^−^ cells were subjected to qRT-PCR and western blot, respectively. CD133 expression is shown by western blot, and *FOXM1* expression is measured using qRT-PCR. **(E and F)** Western blot (with the indicated antibodies) of Ishikawa cells transfected with siRNA against FoxM1 and the control (E). Cell death measured using FACS Canto II analysis with Annexin V-FITC/7-AAD staining, in FoxM1-depleted and control cells, treated with doxorubicin at the indicated dose for 24 hours (F). **(G and H)** Prx3 expression, as determined by western blot, in Ishikawa cells treated with siomycin A, a potent FoxM1 inhibitor (G). The cells were also subjected to sphere formation assays in ultra-low attachment plates. Spheroids were counted two weeks later (H).

Several studies, including our previous study, have reported that Prx3 is upregulated by the transcription factor FoxM1 [17, 23, 30, 31]. Therefore, we measured FoxM1 expression in endometrial cancer tissues and adjacent normal tissues using qRT-PCR. As shown in Figure 7A and 7B, FoxM1 gene showed higher expression in EC tissues than that observed in adjacent normal tissues. In addition, we calculated correlation coefficients to elucidate the relationship between FoxM1 and Prx3 expression (Figure 7C). The correlation coefficient was calculated as 0.55 (P = 0.000443) for Prx3, and a significant positive correlation was observed between FoxM1 expression and Prx3 levels. Moreover, FoxM1 expression was increased in endometrial CSCs isolated from Ishikawa cells (Figure 7D). To determine whether FoxM1 regulates Prx3 expression in Ishikawa cells, we conducted a loss-of-function study using siRNA against FoxM1, and found that Prx3 levels were decreased in FoxM1-depleted cells, which resulted in increased cell death following treatment with doxorubicin (Figure 7E and 7F). Moreover, treatment with siomycin A, a potent specific inhibitor of FoxM1, clearly downregulated Prx3 expression in comparison to that measured in vehicle-treated cells (Figure 7G). Finally, we investigated the tumorigenic ability of FoxM1-depleted cells using a sphere formation assay, and found that the number of spheroids in these cells was reduced by more than 70% compared to that in the control (Figure 7H). Collectively, these results indicate that FoxM1 upregulates Prx3 expression to maintain the survival of endometrial CSCs, by enhancing mitochondrial function, thus promoting tumorigenesis in patients with EC.

## DISCUSSION

The mitochondria are an important target for developing anti-cancer drugs. However, the mitochondrial properties and the mitochondrial-specific targets of endometrial CSCs have not been sufficiently studied. In this study, we found that the expression of mitochondrial Prx3 is upregulated in endometrial CSCs, and that Prx3 plays a critical role in maintaining mitochondrial function via the elimination of ROS, which are produced by the mitochondrial oxidative phosphorylation system. This indicated that mitochondrial Prx3 is important for the survival of CSCs. Furthermore, we found that Prx3 expression is upregulated by the FoxM1 transcription factor in endometrial CSCs. This increase in the expression of Prx3 and FoxM1 contributes to the enhancement of mitochondrial function in endometrial CSCs, compared to endometrial non-CSCs.

Endometrial CSCs have been commonly isolated using an antibody against the CD133 surface marker in EC cells [11, 32–34]. Therefore, we used 10% of these cells (the highly positive and highly negative) from both ends of the CD133^+^ and CD133^−^ populations, to analyze mitochondrial function, and found elevated patterns of mitochondrial function in endometrial CSCs. In a previous study, we reported the mitochondrial and energy metabolism features of colon CSCs, which are often characterized by drug resistance and recurrence. This property correlates with their increased mitochondrial activity and ATP production via oxidative phosphorylation [23]. Moreover, it also was reported that breast CSCs consume more glucose, but produce less lactate and show higher ATP production, compared with controls [35]. Consistent with this, we showed that several mitochondrial properties were specific to endometrial CSCs. In particular, the levels of mitochondrial ROS were increased, as a result of the increased oxidative phosphorylation used to produce ATP in the mitochondria of CSCs (Figure 1). Moreover, we showed that the level of FBP1 was higher in EC tissues than that in the adjacent normal tissues (Figure 1). FBP1 catalyzes the splitting of fructose-1,6-biphosphate into fructose 6-phosphate and inorganic phosphate during gluconeogenesis. Loss of FBP1 increases glucose uptake and glycolysis; conversely, upregulation of FBP1 results in a decrease in glycolysis, and increase in oxidative phosphorylation [28]. Moreover, it was recently reported that cancer cells exhibit diverse responses to glucose limitation, according to cancer type; specifically, mitochondrial oxidative phosphorylation plays a key role in the metabolic process required for the optimal proliferation of some cancer cells under glucose-limiting conditions [36]. Together with these data, our results suggest that EC cells, as well as endometrial CSCs, utilize the primary oxidative phosphorylation pathway, rather than glycolysis, to produce ATP. Therefore, we believe that the mitochondria-dependent ATP production can provide a target for developing CSC-specific anti-cancer drugs, thus promising new strategies for cancer treatment.

ROS are tightly associated with physiological mechanisms governed by the balance between ROS generation and elimination. In particular, the antioxidant peroxiredoxin proteins have been found to differ between stem cells and differentiated cells [37, 38]. Our results also showed that Prx3 is upregulated in endometrial CSCs and in endometrial cancer tissues, compared with its expression in adjacent normal tissues. We also confirmed that Prx3 depletion reduced ATP levels and oxygen consumption in EC cells (Figure 4). In addition, we found that the combination of Prx3 depletion and doxorubicin treatment in endometrial CSCs led to enhanced cell death, which was not observed in CSCs treated with doxorubicin alone (Figure 5). This result indicated that Prx3 eliminated ROS, thus serving as an antioxidant protein. Our sphere and colony formation assays that addressed tumorigenic ability, also revealed that Prx3 depletion combined with doxorubicin treatment caused a decrease in sphere and colony formation, which supported the role of Prx3 (Figure 6). Thus, our data suggest that Prx3, which is upregulated in endometrial CSCs, ensures a proper redox environment to prevent excessive ROS generation and oxidative stress that results from increased mitochondrial oxidative phosphorylation.

We also found that the transcription factor FoxM1 regulates Prx3 expression in endometrial CSCs. Previously, several studies have implicated FoxM1, which is overexpressed in various human malignancies, in tumorigenesis [39, 40]. In addition, it was reported that FoxM1 regulates oxidative stress via the induction of antioxidant proteins such as catalase, superoxide dismutase 2, and Prx3 [31]. Furthermore, we recently reported that FoxM1 binds to the Prx3 promoter in colon cancer [23]. Consistent with these previous studies, our findings showed that FoxM1 knockdown results in a decrease in Prx3 expression, which induces the death of endometrial CSCs (Figure 7). Our results suggest that FoxM1 is a key regulator of Prx3 in endometrial CSCs. Therefore, FoxM1 represents an attractive EC therapeutic target, together with Prx3.

In summary, our study shows that Prx3 and FoxM1 regulate mitochondrial function in endometrial CSCs. The enhanced Prx3 expression maintains mitochondrial function through the elimination of ROS, and the FoxM1-induced Prx3 expression leads to the survival of endometrial CSCs. Our data shows that the sensitized cell death of endometrial CSCs, induced by a combination of Prx3 depletion and doxorubicin treatment, supports the potential utility of this combination treatment, together with mitochondrially targeted compounds, as a promising novel therapeutic strategy for EC.

## MATERIALS AND METHODS

### Cell culture and tissue collection from patients with endometrial cancer

The Ishikawa cell line of human endometrial cancer was obtained from Public Health England (UK). Ishikawa cells were cultured in Minimum Essential Medium α (MEM-α), supplemented with 2 mM glutamine, 1% non-essential amino acids, 5% fetal bovine serum (FBS), and 1% penicillin and streptomycin. Endometrial cancer cell lines were cultured in DMEM/F12, supplemented with 10 ng/mL basic fibroblast growth factor (bFGF) and 20 ng/mL epidermal growth factor (EGF), to obtain spheroids. Cells were cultured at 37°C, in a humidified incubator containing 5% CO_2_. Human endometrial tissue fragments were obtained in accordance with the ethical standards of the Institutional Review Board for human research of the Inje University, Busan Paik Hospital, for human experiments on 25 patients undergoing surgery for endometrial adenocarcinoma. Based on the microscopic features of carcinoma cells, the histological type and grade of the tumors were determined.

### Antibodies and transfection

Antibodies against poly-(adenosine diphosphate-ribose) polymerase, caspase-3, and tubulin were purchased from Cell Signaling Technology (Danvers, MA). Cytochrome *c* and Prx3 antibodies were purchased from BD Pharmingen (San Jose, CA), and Abclone (Seoul, Korea), respectively. The anti-FoxM1 antibody was purchased from Abcam (Cambridge, MA, USA). Antibodies against CD133, including those conjugated to magnetic beads, and allophycocyanin (APC), were purchased from Miltenyi Biotec (Bergisch Gladbach, Germany).

For Prx3 and FoxM1 knockdown, siRNAs (Prx3: 5′-AAGCCAAGTCCAGCTGCTTCC-3′, FoxM1: 5′-CTCTTCTCCCTCAGATATA-3′) were constructed based on the Prx3 and FoxM1 sequences, and exhibited > 80% knockdown efficiencies. For Prx3 overexpression, Ishikawa cells were transfected with 6 μg pCGN-HA or pCGN-HA-Prx3 for 48 hours using Lipofectamine LTX (Invitrogen, Carlsbad, CA) following the manufacturer's protocols, and then doxorubicin was treated with the indicated dosage for 24 hours. The transfected cells were harvested and subjected to western blotting with anti-PARP, anti-HA, and anti-tubulin antibodies.

### Fluorescence-activated cell sorting, magnetic-activated cell sorting, and flow cytometry

Ishikawa human endometrial cancer aggregates were dissociated into single cells, washed with phosphate-buffered saline, and stained with antibodies specific to CD133/1-APC (Miltenyi Biotec). Mouse IgG1 conjugated to APC was used as an isotype control. Cells were sorted using a BD FACS Aria Flow cytometer (Becton Dickinson). Magnetic cell separation was performed on tumor cell populations using microbeads conjugated with CD133/1 (AC133, mouse IgG, cell isolation kit; Miltenyi Biotec). The magnetic separation step was repeated twice using a positive selection column (LS column), followed by a negative selection column (LD column), and then the eluted cells were applied to a new positive selection column. After magnetic sorting, viability was assessed using trypan blue exclusion. The quality of sorting was assessed by flow cytometry, using an antibody against CD133/2 (293C3-phycoerythrin; Miltenyi Biotec) on both CD133^+^ and CD133-depleted cell populations.

### Measurement of mitochondrial activity

To detect and measure the generation of mitochondrial ROS, membrane potential, and Ca^2+^ concentration, we used the specific fluorescent probes Mito-Sox, TMRE, and Rhod-2AM (Invitrogen), respectively. siRNA-transfected cells, and magnetically sorted CD133^+^ and CD133^−^ endometrial cancer cells were cultured and then incubated with 1 mM Mito-Sox for 20 minutes, or with 5 mM TMRE, or Rhod2-AM for 30 minutes at 37°C. The levels of the fluorescent probes were measured using a FACSCantoII flow cytometer (BD Biosciences).

### Measurement of adenosine triphosphate production

To measure the basal and mitochondrial ATP levels, we used the Mitochondrial ToxGlo Assay (Promega, Madison, WI), and followed the manufacturer's protocol. Briefly, CD133^+^ and CD133^−^ cells were plated at 2×10^4^ cells/well in a white-walled, clear-bottom 96-well plate. The plate was centrifuged at 200 × *g* for 3 minutes to remove excess medium. For calculating mitochondrial ATP levels (without mitochondrial materials), 10 mM galactose (to increase cellular oxygen consumption and augment mitochondrial susceptibility), 1 mM rotenone, and 1 mM antimycin A were added to block the activities of the mitochondrial electron transport chain complexes I and III. The plate was incubated at 37°C in a humidified and CO_2_-supplemented incubator for 90 minutes. After reading the plate, ATP levels were measured for each well by adding an ATP detection reagent, and the cells were incubated at 37°C in a humidified and CO_2_-supplemented incubator. The resulting luminescence was quantified using a luminometer (Molecular Devices).

### Measurement of oxygen consumption rate and extracellular acidification rate

To measure the oxygen consumption rate and the extracellular acidification rate, we used an XF24 analyzer (Seahorse Bioscience, Billerica, MA). Briefly, Ishikawa cells were sorted into CD133^+^ subpopulations, and plated at 2×10^4^ cells/well in a Corning Cell Tek (cell-tissue adhesive; Corning, Bedford, MA)-treated XF24 cell culture plate (Seahorse Bioscience). The plate was centrifuged at 200 × *g* for 3 minutes to remove the medium. Then, we added 500 mL of XF Assay Medium (modified DMEM, Seahorse Bioscience), and incubated the plate at 37°C, without CO_2_ for 1 hour. The oxygen consumption rate was measured using the Mito Stress application of the XF24 software, whereas the extracellular acidification rate was measured using the glycolysis application of the XF24 software.

### Quantitation of mitochondrial DNA content

Total DNA, containing chromosomal and mitochondrial DNA, was extracted from cells using the DNeasy kit (Qiagen, Hilden, Germany), following the manufacturer's protocol. The forward and reverse primer sequences were as follows: chromosomal DNA, B2M (forward 5′-CGCGCTACTCTCTCTTTCTG-3′ and reverse 5′-CACCAAGGAGAACTTGGAGA-3′), chromosomal DNA, GAPDH (forward 5′-gaacatcatccctgcctcta-3′ and reverse 5′-TGTCGCTGTTGAAGTCAGAG-3′), mitochondrial DNA, MT-ND2 (forward 5′-GACTATGAGAATCGAACCCATC-3′ and reverse 5′-CCAGGGGATTAATTAGTACGG-3′), and mitochondrial DNA, D-loop (forward 5′-atcaactgcaactccaaagc-3′ and reverse 5′-ACTCTTGTGCGGGATATTGA-3′). Quantitative polymerase chain reaction (qPCR) was performed using the SYBR premix Ex Taq (Takara, Shiga, Japan). All reactions were prepared following the manufacturer's protocol, and were carried out in triplicate. Total DNA was amplified for 60 cycles of 15 seconds at 95°C, 30 seconds at 60°C, and 30 seconds at 72°C for each gene. Expression values are represented relative to the measurements for the B2M locus on chromosomal DNA, in the corresponding samples.

### *In vitro* cell death assays

siRNA-transfected cells were treated with the indicated dose of doxorubicin (Sigma Aldrich, St. Louis, MO) for 24 hours. Magnetically sorted CD133^+^ and CD133^−^ cells were cultured in DMEM/F12 plus supplements (EGF (20 ng/mL), bFGF (10 ng/mL), insulin (25 μg/mL), progesterone (20 nM), estrogen (10 nM), BSA (2 mg/mL), non-essential amino acids (1%), L-glutamic acid (2 mM), apotransferrin (100 μg/mL), putrescine (9.6 μg/mL), and anhydrous sodium selenite (30 nM)), and then the cells were treated for 24 hours with doxorubicin at the indicated dose. Similarly, spheroid cultures were cultured in the presence or absence of doxorubicin in DMEM/F12 plus supplements, after siRNA transfection against Prx3 or control. The viability of cells was evaluated using the Annexin V-fluorescein isothiocyanate (FITC)/7-amino actinomycin D (7-AAD) staining kit (BD Bioscience, San Jose, CA).

### Protein isolation and western blotting

Cells were lysed in lysis buffer A (20 mM N-2-hydroxyethylpiperazine-N0-2-ethanesulfonic acid [pH 7.5], 150 mM NaCl, 1 mM EDTA, 2 mM ethylene glycol tetraacetic acid, 1% Triton X-100, 10% glycerol, and protease cocktail I/II; Sigma), and cellular debris was removed by centrifugation at 10,000 × *g* for 10 minutes. Proteins were separated by sodium dodecyl sulfate polyacrylamide gel electrophoresis, transferred onto nitrocellulose membranes, blocked with 5% skim milk in 0.01 M TBS (pH 7.5), containing 0.5% Tween 20, and blotted with the appropriate primary antibodies. The antigen-antibody complexes were detected by chemiluminescence (Abclone, Korea).

### Quantitative reverse transcriptase polymerase chain reaction

Total RNA from human endometrial tumor tissues, non-tumor tissues, and CD133^+^ and CD133^−^ cells was extracted using Trizol (Invitrogen). RNA (1 μg) was reverse transcribed using the First-Strand cDNA Synthesis Kit (Fermentas, Grand Island, NY). All reactions were performed in triplicate, and B2M was used as a control. Using the comparative threshold cycle (Ct) method or the standard method, the relative quantification of gene expression was calculated as the ratio of CD133^+^ to CD133^−^ cells after normalizing against B2M for each sample. The forward and reverse primer sequences were as follows: Prx3 (Forward 5′-GTTGTCGCAGTCTCAGTGGA-3′ and reverse 5′-GACGCTCAAATGCTTGATGA-3′), FoxM1 (Forward 5′-GGAGGAAATGCCACACTTAGC-3′ and reverse 5′-TGTAGGACTTCTTGGGTCTTGG-3′), and B2M (Forward 5′-CTCGCTCCGTGGCCTTAG-3′ and reverse 5′-CAAATGCGGCATCTTCAA-3′).

### Colony-forming assay and sphere-formation culture

Anchorage-independent growth was assessed by performing colony-forming assays in soft agar, and sphere formation cultures. For colony-forming assays, cells were suspended in 1 mL cell growth medium without FBS containing 0.3% agar, and plated over a layer of 0.6% agar in growth medium without FBS. Cells were grown at 37°C in 5% CO_2_, and then 15 days post-inoculation, the colonies were stained with 0.01% Crystal Violet (Sigma) for 10 minutes, and counted. For sphere formation, cells were seeded in ultra-low-attachment 6-well plates at a density of 10^3^ cells/mL. Sphere cultures were grown in serum free DMEM/F-12 containing supplements.

### Migration assay

The migration assay was performed with a 24 well Transwell culture chamber (BD Bioscience, San Jose, CA; 8 μm pore size). The bottom side of the filter was coated with gelatin B (1μg/μl) and air dried for 1hr. The filter was placed in the chamber wells filled with MEM-α, supplemented with 2 mM glutamine, 1% non-essential amino acids, 5% FBS. Serum-starved cells (2 × 10^4^) were added on top of each filter and the chamber was incubated at 37°C in 5% CO_2_ incubator for 24 hrs. The non-migrated cells were removed from the top of the membrane, and then the migrated cells were stained with hematoxylin for 15 minutes at room temperature after incubation at 0.5% Triton X-100 for 3 min.

### Statistical analysis

Data were analyzed using the Student's *t* test with SigmaPlot 12.0 software (2013, Systat Software Inc., San Jose, CA). P values were derived to assess statistical significance, and are indicated as follows: ^*^P < 0.05; ^**^P < 0.01; and ^***^P < 0.001. Data for all figures are expressed as the means ± SDs of three independent experiments.
